# Supplementary figures and images for: Long-COVID in children and adolescents: a systematic review and meta-analyses
Source: Sci Rep. 2022 Jun 23;12:9950. doi: 10.1038/s41598-022-13495-5 (PMC9226045; doi:10.1038/s41598-022-13495-5)

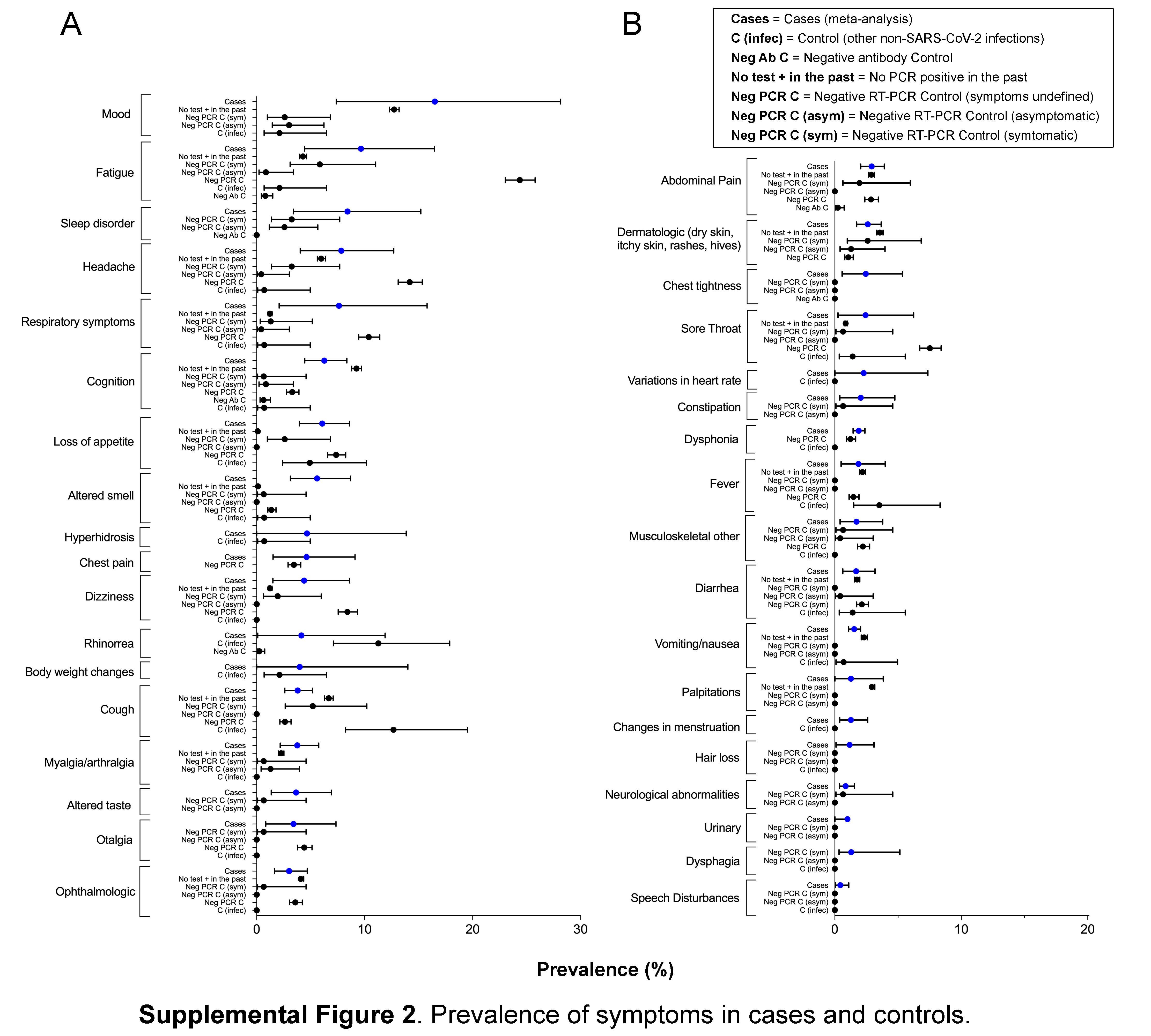

Supplement: Supplementary file 2 — Supplementary Figure 2. [file 41598_2022_13495_MOESM2_ESM.jpg]
